# Supplementary material for: Integrated impedance sensing of liquid sample plug flow enables automated high throughput NMR spectroscopy
Source: Microsyst Nanoeng. 2021 Apr 14;7:30. doi: 10.1038/s41378-021-00253-2 (PMC8433180; doi:10.1038/s41378-021-00253-2)
Supplement: Supplementary file 1 — Supplemental Material [file 41378_2021_253_MOESM1_ESM.pdf]

Integrated impedance sensing of liquid sample plug flow enables automated high throughput NMR spectroscopy

Omar Nassar[1], Mazin Jouda[1], Michael Rapp[1], Dario Mager[1], Jan G. Korvink[1], and Neil MacKinnon*[1]

[1]*Institute of Microstructure Technology, Karlsruhe Institute of Technology (KIT), Hermann-von-Helmholtz-Platz 1, 76344 Eggenstein-Leopoldshafen, Germany*

Supplementary information

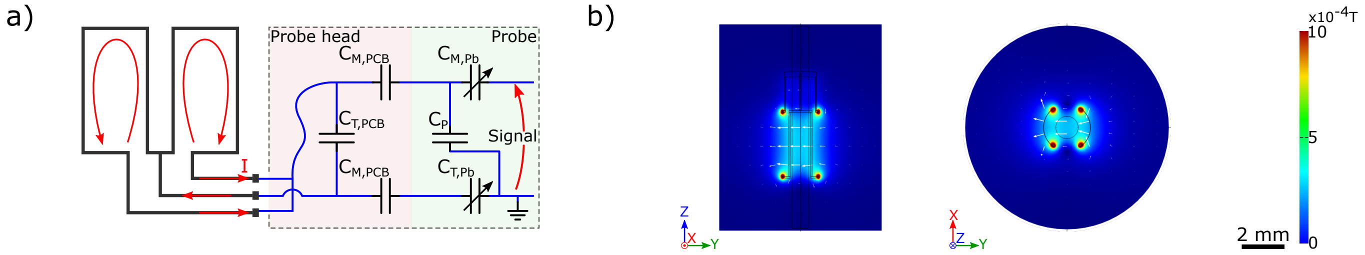

Figure S1: RF connection and field performance of saddle coil. a) The electrical connection of the NMR saddle detector. The three-terminals of the saddle detector are connected in a way to keep the current flowing in the opposite direction in each loop. Hence, the two magnetic fields will constructively interfere inside the coil (sample region). b) The simulated magnetic B_1 fields of the two loops are pointing in the same direction.

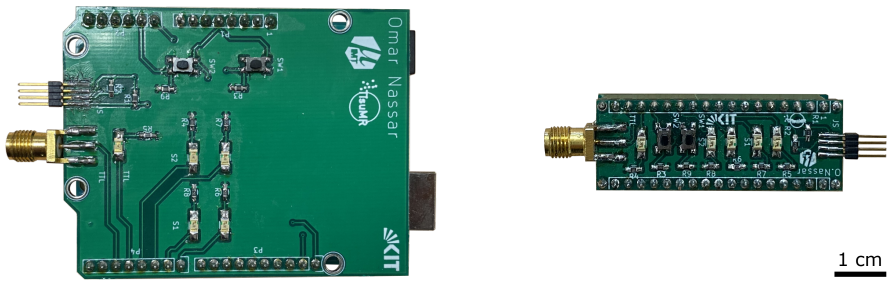

Figure S2: Two Arduino shields were fabricated and used for the NMR experiments. One shield compatible with Arduino Uno (left), and the other shield compatible with Arduino micro (right). The performance of the microcontrollers was equivalent.

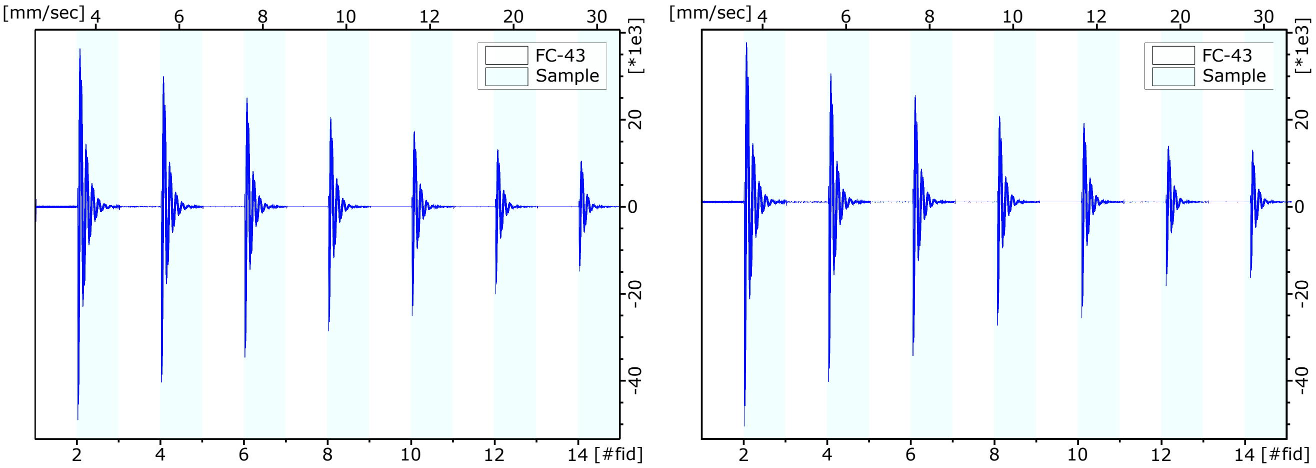

Figure S3: Flow rate control while performing the flow NMR experiment can be done manually (right) or automatically (left). In both cases, no noticeable effect on the spectrum was observed. In the automatic control case, the syringe pump (Fusion 200-X Touch, KR Analytical) was used, controlled via LabVIEW software over a Universal Serial Bus (USB) connection. In this experiment, the capacitance signal was used to update the flow rate in real-time, i.e., after the NMR signal acquisition of an aqueous sample was completed, the same trigger signal sent to the spectrometer used to save the FID was also used to instruct the pump to change the flow rate.

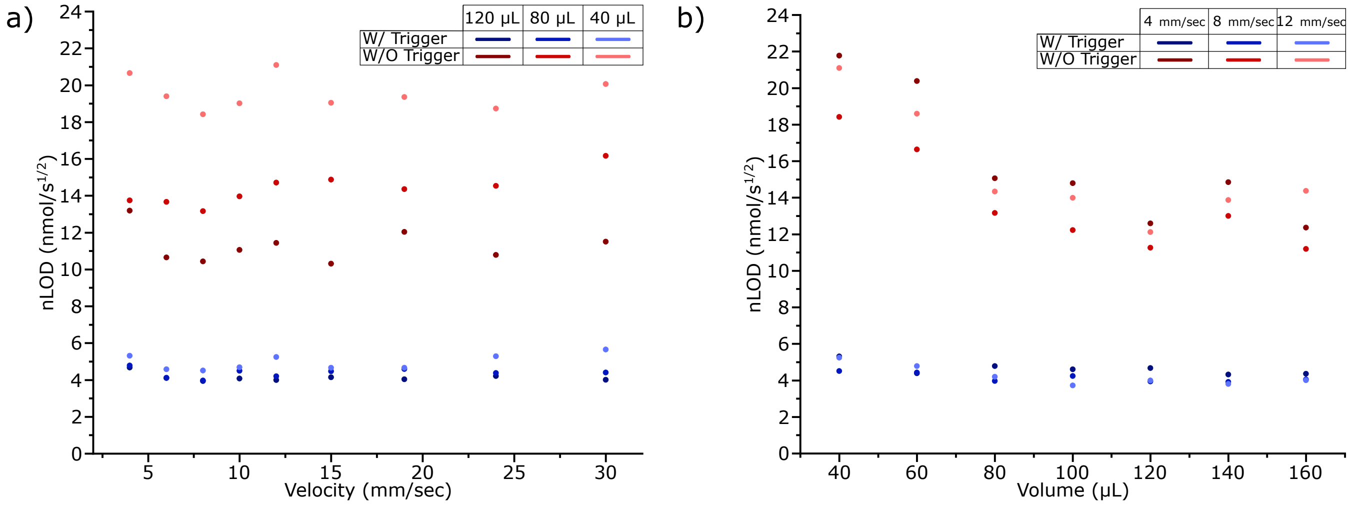

Figure S4: Sensitivity results under varying flow rates and sample volumes. a) The normalized limit of detection ($nLOD_\omega$) versus sample velocity. The $nLOD_\omega$ varies within a small range over the enter range of velocities for a given volume. Under manual triggering conditions, the $nLOD_\omega$ increases as the sample volume decreased due to the increased contribution of the oil signal to the FID (noise). b) The normalized limit of detection ($nLOD_\omega$) versus sample volume. Under auto-triggering conditions, a minimal enhancement in the $nLOD_\omega$ is achieved by increasing the sample volume. On the other hand, in manual triggering, the $nLOD_\omega$ is highly enhanced by increasing the sample volume as the signal contribution from the oil plugs (i.e. noise) is reduced. The mean $\pm$ standard deviation of these data are plotted in Fig. 4, main text.

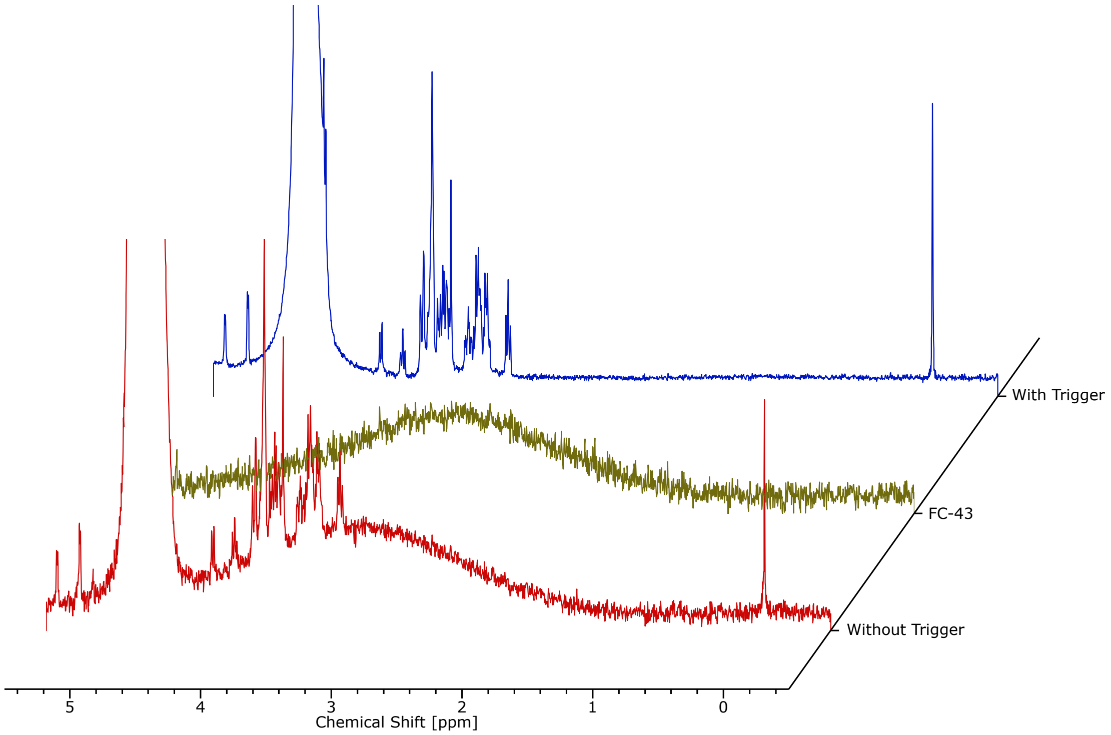

Figure S5: Representative ^{1}H NMR spectra. The blue spectrum is of a 120 µL sample plug (300 mM glucose, 75 g L^{-1} coloring powder, and 30 mM TSP dissolved in DI water) acquired using the auto triggering system. In contrast, the red spectrum of the same sample obtained with manual triggering. The flow velocity was 4 mm s^{-1}.

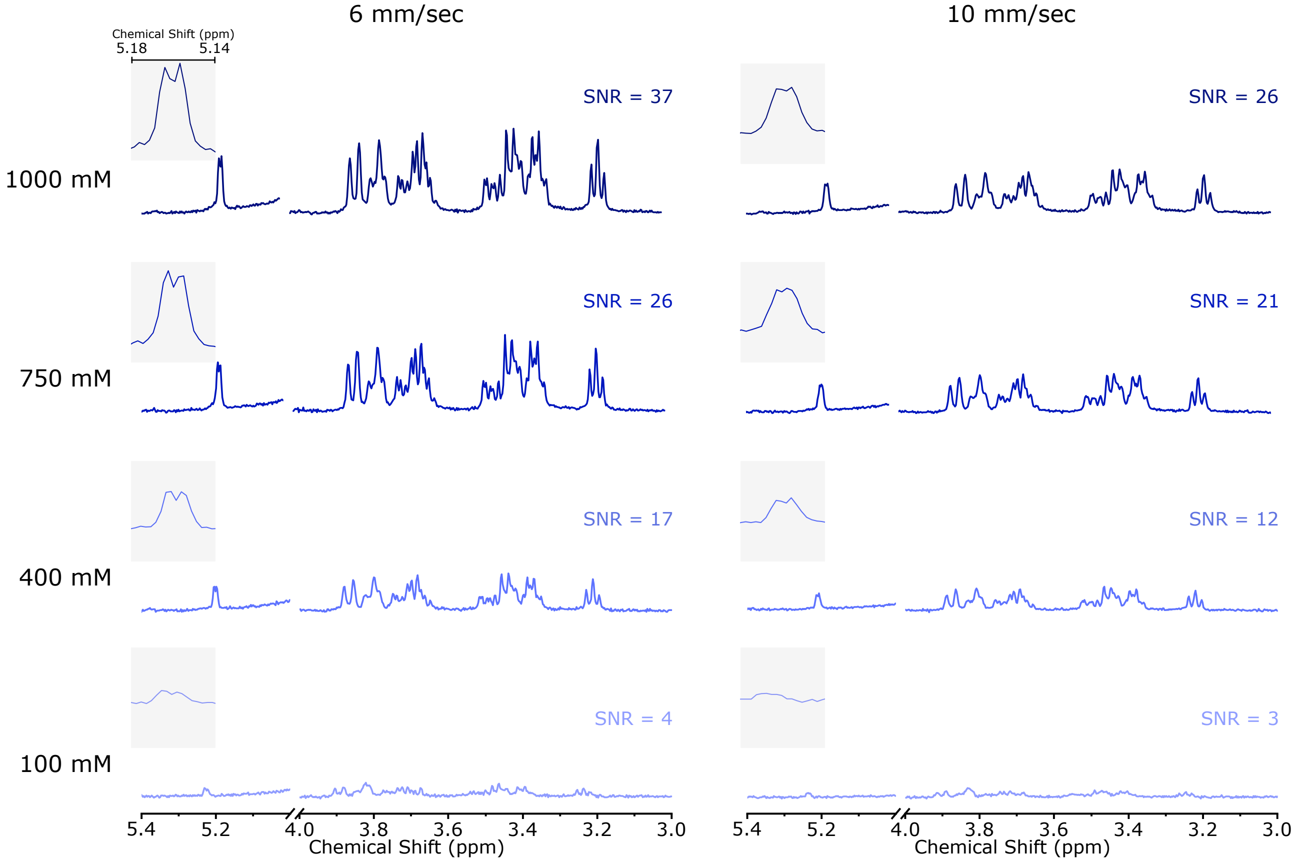

Figure S6: Selected ^{1}H NMR spectra from the flow NMR spectroscopy experiments varying the glucose concentrations in DI water. The SNR of the glucose anomeric proton for each spectrum is noted next to the signal with an expanded view of the peak (5.18-5.14 ppm) for spectral resolution comparison. The spectral region between 4-5 ppm (water resonance) was excluded for clarity.

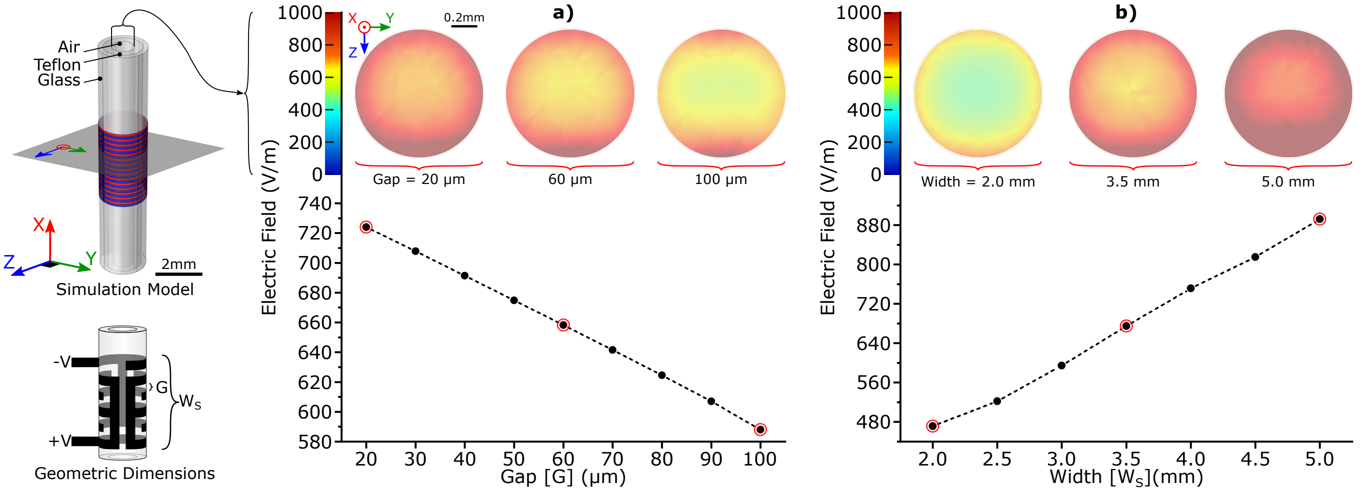

Figure S7: Finite element simulation (COMSOL Multiphysics) determine the effect of changing the gap between the capacitive sensor's fingers (G) and the total sensor's width (W_S) on the generated electric field inside the sensor. a) Gap dependence: (top) top views of the electric field distribution within the sample region are shown in the cases of gaps of 20 μm, 60 μm, and 100 μm; (bottom) the plotted values of the electric fields are computed at the center of the sensor. The electric field decreases with increasing the gap between the fingers. b) Width dependence: (top) top views of the electric field distribution are shown for sensors with widths of 2 mm, 3.5 mm, and 5 mm; (bottom) the electric field increases with increasing the sensor width.

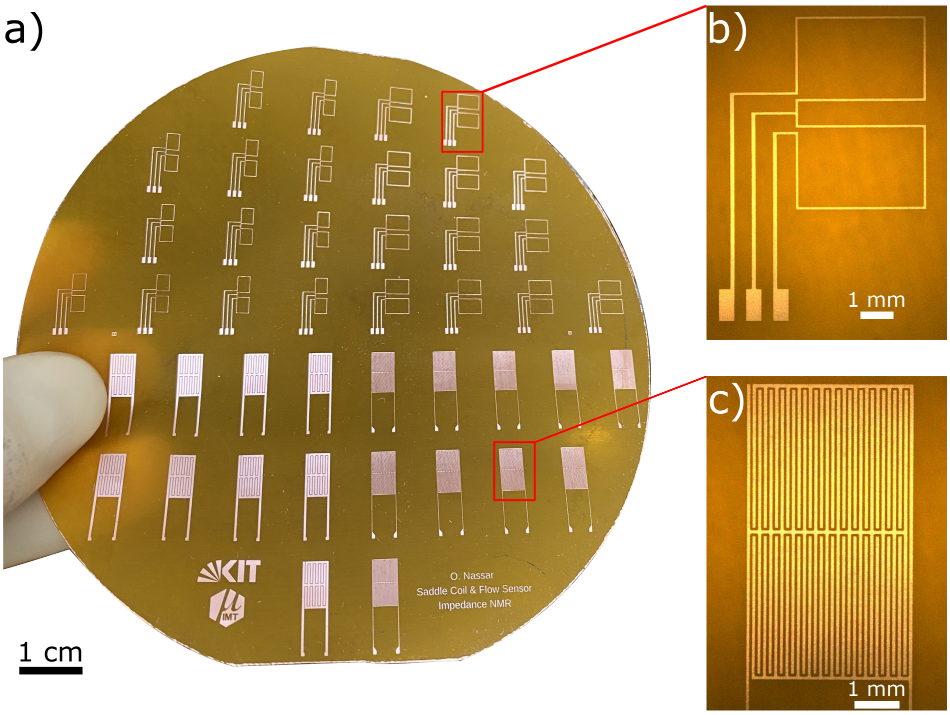

Figure S8: Representative microfabrication results. a) A photograph of a fully patterned Kapton film in the shape of a 100 mm wafer. The film possesses a batch of the micro saddle NMR detector and the interdigitated capacitive sensor, demonstrating the developed process's compatibility with mass production. The sub-figures show microscopic images of the 2D patterned (b) saddle detector and (c) interdigitated capacitive sensor.
